# Complete Resolution of New Daily Persistent Headache With Migraine-Like Features Following Erenumab Treatment: A Case Report

**DOI:** 10.7759/cureus.84442

**Published:** 2025-05-19

**Authors:** Shoji Kikui, Daisuke Danno, Takao Takeshima

**Affiliations:** 1 Department of Neurology, Headache Center, Tominaga Hospital, Osaka, JPN

**Keywords:** calcitonin gene-related peptide (cgrp), cgrp receptor antagonist, chronic migraine, continuous daily headache, erenumab, migraine-like features, monoclonal antibody therapy, new daily persistent headache, phenotypic stratification, primary headache disorder

## Abstract

New daily persistent headache (NDPH) is a rare and difficult-to-treat primary headache disorder characterized by a sudden onset of continuous, unremitting pain. We report the case of a 46-year-old Japanese woman with a history of episodic migraine who developed a persistent daily headache with migraine-like features. Despite multiple conventional preventive treatments, her symptoms remained unchanged. Remarkably, she achieved complete and sustained remission after initiating monthly subcutaneous injections of erenumab at a dose of 70 mg. The treatment was maintained for 24 months, during which no recurrence of the continuous headache occurred. This case highlights the diagnostic overlap between NDPH and chronic migraine presenting with continuous daily headache and suggests the potential benefit of calcitonin gene-related peptide-targeted therapy in patients with migraine-like NDPH phenotypes. Careful phenotypic characterization may improve therapeutic decision-making and support a syndromic approach to managing continuous daily headache disorders.

## Introduction

New daily persistent headache (NDPH) is an idiopathic primary headache syndrome characterized by the abrupt onset of a continuous, unremitting daily headache that does not evolve from a prior pattern of escalating attacks and cannot be attributed to any other primary or secondary headache disorder [1]. First described in 1986 and historically reviewed by Robbins et al. in an interview with Vanast [1], NDPH has since been recognized as a heterogeneous clinical entity with features that frequently overlap with chronic migraine and chronic tension-type headache [2].

According to the International Classification of Headache Disorders, 3rd edition (ICHD-3), NDPH is defined by the following criteria [2]: (A) persistent headache meeting criteria B and C; (B) a distinct and clearly recalled onset, with pain becoming continuous and unremitting within 24 hours; (C) a duration of more than three months; and (D) exclusion of other ICHD-3 diagnoses.

Despite its formal inclusion in ICHD-3, the underlying nature of NDPH remains poorly understood. Recent perspectives, particularly those proposed by Goadsby, argue that NDPH should be considered a clinical syndrome rather than a single disease entity, encompassing a range of phenotypes that may reflect distinct underlying mechanisms [3].

Managing NDPH is notably difficult, as patients often show poor response to standard preventive treatments for migraine, resulting in long-term impairment of quality of life [4]. Some patients diagnosed with NDPH exhibit migraine-like features, such as unilateral, pulsating pain, photophobia, phonophobia, nausea, and a personal or family history of migraine, suggesting the existence of a “migraine-like NDPH” phenotype. This subset may respond differently to treatment, particularly to therapies targeting the calcitonin gene-related peptide (CGRP) pathway.

Current treatment strategies are mostly empirical and include drugs commonly used for chronic migraine and tension-type headache, such as tricyclic antidepressants, antiepileptic agents (e.g., valproate), beta blockers, and calcium channel blockers [4]. However, the effectiveness of these treatments is often limited [4]. Short courses of corticosteroids [4] and trials with indomethacin, to rule out hemicrania continua, may also be employed [2]. Prognosis varies widely: while some patients achieve spontaneous remission within a few months, many experience persistent symptoms lasting for years, often with significant functional impairment [4]. Prognosis tends to be especially poor in patients with prominent migraine-like features.

Recent evidence implicates CGRP in the pathophysiology of a subset of NDPH cases with migraine-like characteristics [5], suggesting that CGRP-targeted therapies may offer both therapeutic benefit and diagnostic insight.

This report describes a patient initially diagnosed with NDPH with migraine-like features who experienced complete and sustained remission following treatment with erenumab, a CGRP receptor antagonist. The case illustrates the diagnostic complexity of distinguishing NDPH from chronic migraine with continuous daily headache and emphasizes the potential value of phenotypic stratification in guiding individualized treatment strategies.

## Case presentation

A 46-year-old Japanese woman presented with the sudden onset of a severe headache localized to the right temple. Her medical history was notable for pulsatile headaches in the right orbital and temporal regions, accompanied by nausea and phonophobia, occurring approximately once per month since her 30s. She had previously been diagnosed with episodic migraine without aura and had no history of acute medication overuse. There was no history of other medical conditions, alcohol consumption, or smoking. Her family history was significant for migraine in her sister and niece and Alzheimer’s disease in her father.

On the day following the headache onset, the patient visited the ED of another hospital. Brain MRI and magnetic resonance angiography revealed no structural abnormalities. Despite the administration of intravenous antiemetics and acetaminophen, her headache persisted. She was subsequently referred to our Headache Center and admitted for further evaluation.

On admission, the patient was alert, and cardiopulmonary findings were unremarkable. Her vital signs were stable. No vesicular skin lesions suggestive of varicella zoster virus infection were observed, and neurological examination, including assessment for nuchal rigidity, was normal. She described the headache as a moderate-to-severe pulsatile pain in the right orbital and temporal areas, accompanied by photophobia, phonophobia, and nausea during intense episodes. Her numeric rating scale (NRS) score reached 10 at peak intensity.

Laboratory investigations, including inflammatory markers, autoimmune screening, and thyroid function tests, were within normal ranges. Cerebrospinal fluid analysis ruled out meningitis and intracranial hypotension syndrome. Repeat brain MRI, magnetic resonance angiography, and magnetic resonance venography were performed to investigate potential secondary headache disorders. Conditions such as subarachnoid hemorrhage, cerebral venous thrombosis, arterial dissection, and reversible cerebral vasoconstriction syndrome were excluded.

Sumatriptan (50 mg, as needed) and rizatriptan (10 mg, as needed) were ineffective during acute attacks, and diclofenac (25 mg, as needed) provided only mild relief. Preventive treatments for migraine - including lomerizine (20 mg daily), valproate (400 mg daily), and propranolol (60 mg daily) - were initiated but failed to alleviate either her continuous baseline headache (NRS 7) or the severe intermittent exacerbations (NRS 10) that occurred several times daily. Considering the possibility of hemicrania continua, acemetacin (180 mg daily), an indomethacin prodrug, was administered, but it led to minimal improvement. A short-term course of intravenous corticosteroids (hydrocortisone 300 mg/day for seven consecutive days) was also ineffective.

After two months of hospitalization, her continuous headache remained unchanged, although the frequency of severe episodes had slightly decreased. She was discharged and scheduled for outpatient follow-up. Three months after onset, her headache remained stable, and she voluntarily discontinued prophylactic medications without worsening of symptoms. However, she was unable to return to work and continued to convalesce at home due to persistent functional impairment. A diagnosis of NDPH (code 6.6.1) was made based on the ICHD-3 criteria. Given the prominent migraine features and her history of episodic migraine, a migraine-like phenotype within the clinical syndrome of NDPH was suspected.

Given the significant functional impairment and the phenotypic profile suggestive of a CGRP-related mechanism, treatment with erenumab, a CGRP receptor antagonist, was initiated based on previous reports of its efficacy in similar migraine-like NDPH phenotypes. Her headache resolved completely following the first dose of erenumab. She continued to receive monthly subcutaneous injections of erenumab at a dose of 70 mg throughout two years of follow-up. During this period, she experienced only mild monthly migraine-like attacks, which were successfully managed with diclofenac. No recurrence of persistent headache or erenumab-related adverse events was observed.

## Discussion

This case underscores the diagnostic challenges associated with continuous daily headache presenting with migraine-like features. Although the patient initially met the diagnostic criteria for NDPH according to the ICHD-3, her complete and sustained remission following treatment with erenumab suggests that the underlying disorder may have been a continuous form of chronic migraine.

The patient’s history of episodic migraine without aura, unilateral pulsatile pain, photophobia, phonophobia, and nausea during severe episodes, along with a positive family history of migraine, strongly supports a migraine-related pathophysiology. Her notable response to a CGRP receptor antagonist further aligns with recent evidence. Bancalari et al. reported the successful use of CGRP monoclonal antibody therapy in patients diagnosed with NDPH exhibiting migraine-like features, suggesting the therapeutic potential of this approach [5]. Additionally, a real-world study by Buture et al. showed that approximately one-third of patients with abrupt-onset, unremitting, treatment-refractory headaches with migraine-like features experienced significant improvement with erenumab [6].

These findings highlight not only the therapeutic value of CGRP-targeted agents in this difficult-to-treat population but also the importance of early recognition of migraine phenotypes to guide treatment selection and improve clinical outcomes in patients initially diagnosed with NDPH-like continuous headaches [5,6].

Distinguishing migraine-like NDPH is clinically meaningful, as it implies a potential overlap with the pathophysiology of chronic migraine and may justify the use of migraine-specific treatments. In particular, the observed responsiveness to CGRP-targeted therapy in this case suggests a mechanistic convergence that reinforces the utility of phenotypic stratification in clinical practice [3-6].

While the pathophysiology of NDPH remains incompletely understood, several hypotheses have been proposed. One theory posits that NDPH results from a maladaptive response to a triggering event, such as a viral infection, psychological stress, or minor head trauma, leading to persistent activation of the trigeminovascular system [3,4]. Neuroinflammatory mechanisms, including glial cell activation and proinflammatory cytokine release, may contribute to the maintenance of chronic pain. Recent neuroimaging studies have revealed abnormalities in brain regions involved in pain modulation, such as the periaqueductal gray and thalamus [4]. In some patients, NDPH may share overlapping mechanisms with chronic migraine, particularly when migraine-like features are prominent. The involvement of the CGRP pathway in such cases is supported by clinical responses to CGRP-targeted treatments, as demonstrated in our patient [5,6].

**Figure 1:**
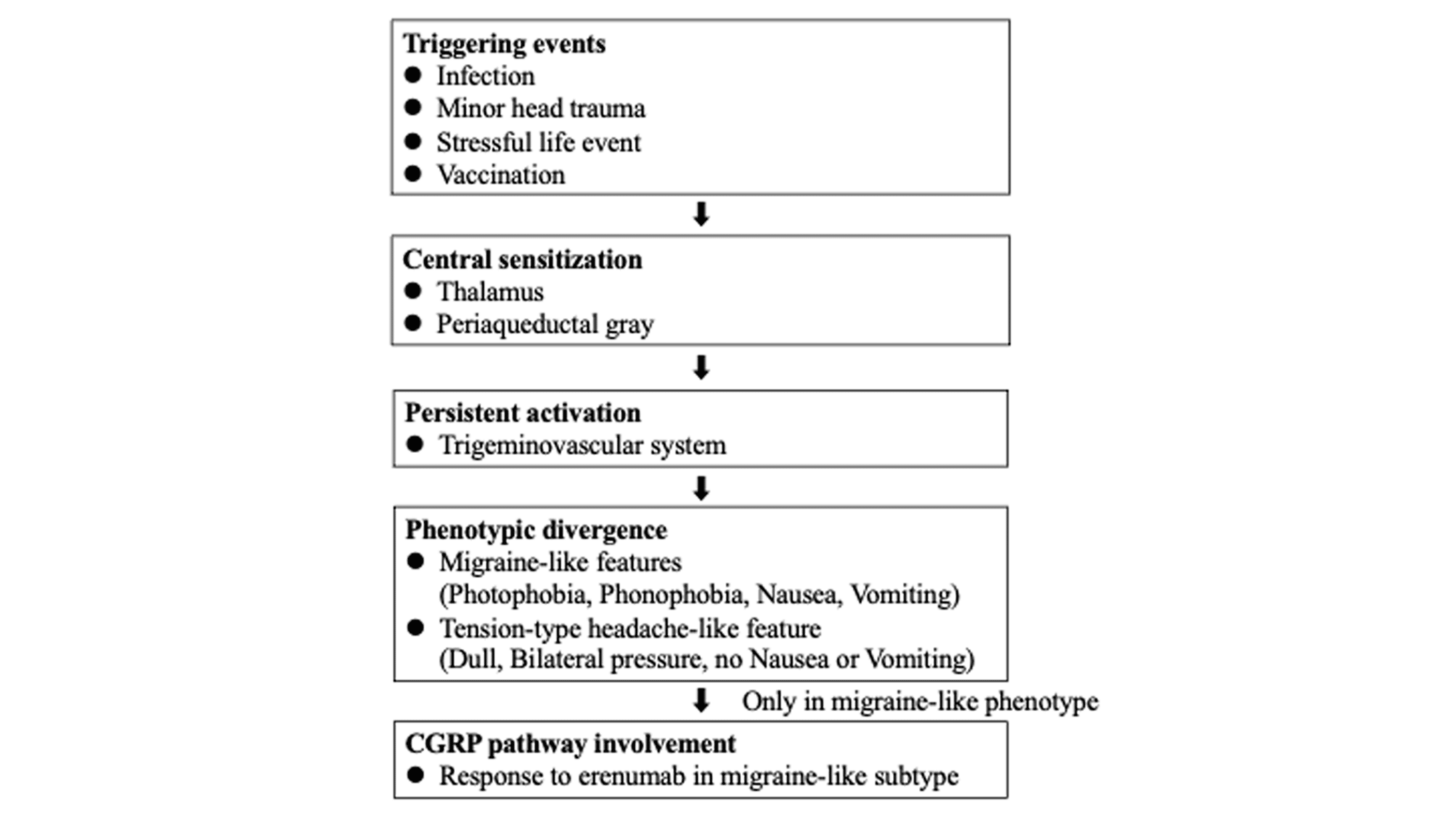
Proposed mechanisms underlying NDPH and its migraine-like phenotype This conceptual model illustrates the proposed pathophysiological mechanisms of NDPH. Potential triggers, such as infections or stress, may initiate trigeminovascular activation and central sensitization. These processes can give rise to divergent clinical phenotypes. In cases with migraine-like features, involvement of the CGRP pathway may account for the observed responsiveness to CGRP-targeted therapies. CGRP, calcitonin gene-related peptide; NDPH, new daily persistent headache

Figure 1 presents a conceptual model outlining the proposed mechanisms underlying NDPH, particularly in migraine-like phenotypes. The model emphasizes the contribution of trigger factors, trigeminovascular activation, central sensitization, neuroinflammation, and CGRP pathway involvement - factors that collectively drive chronicity and resistance to treatment.

As originally proposed by Goadsby in 2011 [3] and supported by subsequent studies [4], NDPH may be more appropriately conceptualized as a clinical syndrome encompassing a spectrum of phenotypes rather than as a single disease entity. This case aligns with that perspective, given the patient’s migraine-like features and favorable response to CGRP-targeted therapy. Within this framework, patients presenting with abrupt-onset, persistent headaches should be phenotypically stratified based on their clinical background, particularly in relation to prior migraine history and symptom profile.

Alternatively, while the patient’s presentation was suggestive of chronic migraine with continuous daily headache, the possibility of a CGRP-sensitive subset within primary NDPH cannot be entirely ruled out. This consideration highlights the diagnostic complexity and overlap between NDPH and chronic migraine, underscoring the need for a flexible, phenotype-driven diagnostic approach informed by both symptomatology and treatment response.

In Japan, erenumab is approved for the “prevention of migraine attacks.” In this case, given the patient's history of episodic migraine, continuous migraine-like symptoms, and sustained response to CGRP-targeted therapy, her clinical presentation was considered to share characteristics of both NDPH and chronic migraine with continuous daily headache. Therefore, the use of erenumab was deemed clinically appropriate.

## Conclusions

This case illustrates that erenumab, a CGRP receptor antagonist, can be an effective treatment for patients with continuous daily headache exhibiting migraine-like features, even when initially diagnosed with NDPH. Careful clinical phenotyping - taking into account prior migraine history and symptom characteristics - may help differentiate between NDPH and chronic migraine and inform targeted treatment strategies. These findings support a syndromic, rather than purely categorical, approach to managing persistent daily headache disorders.
